# The effect of different exercise training modes on improving quality of life in patients with Parkinson's disease: a network analysis

**DOI:** 10.3389/fneur.2025.1601080

**Published:** 2025-07-02

**Authors:** Ying Li, Ruixin Zhuang, Jianhua Zhang, Xiaojie Liu

**Affiliations:** ^1^College of Sports Science, Jishou University, Jishou, Hunan, China; ^2^School of Physical Education and Arts, Hunan University of Medicine, Huaihua, Hunan, China; ^3^Faculty of Health Sciences and Sport, Macao Polytechnic University, Macau, Macao SAR, China; ^4^Welfare Center (Elderly Care Service Department), Shanghe County Hospital of Traditional Chinese Medicine, Jinan, Shandong, China

**Keywords:** quality of life, systematic review, Parkinson's disease, meta-analysis, system review

## Abstract

**Objective:**

Reduced quality of life is a typical manifestation of Parkinson's disease (PD) patients, and exercise has a significant effect on improving the quality of life of PD patients. However, it is still unclear which exercise is effective in improving quality of life for people with Parkinson's disease. The aim of this study was to compare effective exercises for improving quality of life in patients with Parkinson's disease through a network meta-analysis.

**Method:**

We conducted comprehensive database searches, including PubMed, Cochrane Library, Embase, Web of Science and CNKI. The included studies assessed methodological quality using the Cochrane Bias risk tool, and we collected information from the studies to compare the effects of 25 exercise interventions on quality of life in PD patients.

**Results:**

The results of the network meta-analysis showed that QG is higher than OE (MD, −9.26; 95%CI, −18.25 to −0.27), FAE (MD, −10.77; 95%CI −19.52 to −2.02), VR (MD, −10.65; 95%CI −19.70 to −1.60), TC (MD, −11.06; 95%CI −21.32 to −0.81), CT (MD, −11.42; 95%CI −20.73 to −2.11), RT (MD, −11.60; 95%CI −20.72 to −2.49), CE (MD −12.50; 95%CI −24.47 to −0.52), GT (MD −13.10; 95%CI −24.67 to −1.52) to better improve quality of life in patients with PD. DE is superior to TR (SMD, −2.10; 95%CI −3.75 to −0.45), BT (SMD, −2.63; 95%CI −4.93 to −0.32), BDJ (SMD, −4.14; 95%CI −6.15 to −2.14), RT (SMD, −4.54; 95%CI −6.94 to −2.14), TC (SMD, −5.28; 95%CI −7.73 to −2.84) in reduce depression. ROT is superior to TR (MD, 6.17; 95%CI 0.57 to 11.77), in improving balance capacity.

**Conclusion:**

Our study found that QG improved the quality of life in patients with PD better than other forms of exercise. DE is more effective than other exercises in reducing depression in PD patients. ROT is better at improving the balance of PD patients.

## Introduction

Parkinson's disease (PD) is a common neurodegenerative disorder primarily caused by an imbalance of neurotransmitters and a depletion of dopamine in the brain (1). Growing evidence indicates that the incidence of PD has increased in recent years, with reported rates ranging from 3.5 to 42.8% (2). Patients with PD typically present motor symptoms such as progressive motor retardation, myotonia, and abnormal posture and gait. These motor symptoms are often accompanied by non-motor symptoms such as depression, impulse control disorders, anxiety, and insomnia (3). Compared with motor symptoms, non-motor symptoms in patients with PD are more strongly associated with pain severity and hospitalization rates (4). Among these non-motor symptoms, depression and anxiety are common psychiatric conditions that substantially affect the quality of life and contribute to negative psychological states in patients with PD (5). In addition, factors such as self-image, life satisfaction, and social interactions are often negatively affected, which in turn further reduces patients' overall quality of life (6). The World Health Organization (WHO) defines quality of life as “an individual's perception of their position in life in the context of the culture and value systems in which they live and in relation to their goals, expectations, standards, and concerns (7).” Quality of life encompasses physical, psychological, cognitive, and environmental factors, as well as autonomy and social relationships (7, 8). The primary goal of PD treatment is to minimize its negative impact on the patient's functional abilities and overall quality of life. Evaluating how the disease affects quality of life serves as a key indicator of treatment effectiveness (9).

At present, the treatment of Parkinson's disease includes drug therapy DBS surgery (deep brain stimulation) (10), while Western medicine mainly focuses on drugs such as levodopa and dopamine receptor agonists. Drug therapy can temporarily improve the physical disability of patients and control some symptoms through the replacement of dopamine in the brain. However, PD patients taking drugs for a long time will lead to side effects such as depression, anxiety, delusion, hallucination, and even mental disorders, and reduce the quality of life of PD patients. Exercise has been identified as a possible adjunctive treatment for Parkinson's disease, and periodic exercise can effectively improve motor function, depressive symptoms, sleep disturbances, and quality of life in patients (11). In one study, Ferreira et al. (12) found that resistance training was effective in improving the quality of life of PD patients. Moratelli et al. (13) found in a study that Brazilian dance can effectively improve the quality of life of PD patients. In a systematic review and meta-analysis, Song et al. (14) found that Tai Chi and Qigong mind-body exercises effectively improved the quality of life in patients with PD. At present, there is still controversy about what kind of exercise can effectively improve the quality of life of PD patients.

Network meta-analysis (NMA) has become increasingly common for evaluating medical interventions because it estimates the relative effectiveness of all interventions and the ranking of interventions, even in the absence of direct dairy comparisons (15).

However, the current NMA on the improvement of exercise in patients with Parkinson's disease mainly focuses on the movement symptoms of PD patients (16, 17), and there is a lack of research on the quality of life of PD patients. Our study took the quality of life of PD patients as the primary outcome indicator, compared the impact of 25 different exercises on the quality of life of PD patients, determined the best exercise mode to improve the quality of life of PD patients, and guided PD patients to choose the best exercise mode.

## Methods and analysis

### Registration

This network meta-analysis was designed according to the guidelines for Preferred Reporting Items of Systems Review and Network Meta-Analysis (PRISMA-NMA) (18).

### Search strategy

The computer searched PubMed, Web of Science, Embase, Cochrane Library, CNKI, and other databases, and the search period was established until August 20, 2024. The search takes the way of combining subject words and free words. The search strategy uses Pubmed as an example, as shown in Appendix 1.

### Study selection

The inclusion criteria for study selection were based on the PICOS methodology (Participants, interventions, comparators, outcomes, and study design) (18), see Table 1.

**Table 1:** Inclusion criteria and exclusion criteria.

**Category**	**Inclusion criteria**	**Exclusion criteria**
Population	Parkinson's disease was diagnosed in patients > 18 years of age	Patients with severe comorbidities such as severe hypertension, heart disease, or other serious systemic diseases
Interventions	Aquatic Exercise (AQE), Whole body vibration training (WBV), Virtual reality (VR), Treadmill training (TT), Resistance training (RT), Tai Chi (TC), Biofeedback Balance and Gait Training (BGT), Dance exercise (DE), Balance training (BT), Game training (GT), Baduanjin (BDJ), Home exercise (HE), Yoga (YG), Combined therapy (CT), Stretch exercise (SE), Five animal exercises (FAE), Other exercise (OE), Fitness exercise (FE), Qigong (QG), Virtual reality balance training (VRB), Cycling exercise (CE), Robotic Training (ROT), Core strength training (CST).	
Comparisons	Traditional Rehabilitation (TR), Control group (CON)	
Outcomes	Main outcome measures: quality of life Secondary outcome measures: depression, balance capacity	
Study	Randomized controlled trial; published in English or Chinese	

Each intervention is defined in Appendix 2.

### Data extraction

The following data were extracted independently by two reviewers: first author, year of publication, country, sample size, intervention mode, intervention time, and intervention period. Data are expressed as mean ± standard deviation (mean ± SD). If the outcome indicator reports multiple points, we extract data for the most recent time.

### Risk of bias assessment

The risk of bias was assessed independently by two reviewers and by a third reviewer using the tools provided by the Cochrane Collaboration (19), including sequence generation, hidden assignment, blinking, incomplete outcome data, non-selective reporting of results, and other sources of bias. Each criterion was judged to have a low, unclear, or high risk of bias.

### Data analysis

The netmeta package of R-4.2.1 software was used to perform mesh meta-analysis. Use the STATA 15.1 “networkplot” feature to draw and generate a network diagram that describes and presents different forms of exercises. We use nodes representing various interventions and edges representing head-to-head comparisons between interventions. Node splitting assesses inconsistencies between direct and indirect comparisons (20). The combined estimates and their 95% confidence intervals (95%CI) were calculated using random effects network analysis. When we are interested in results using the same unit of measure in a study, consider analyzing the results as a therapeutic effect by means difference (MD) or evaluating standardized mean difference (SMD). A pair-to-pair random-effects meta-analysis was used to compare various exercise therapies. The heterogeneity of all pair-to-pair comparisons was assessed using the *I*^2^ statistic, and publication bias was evaluated using the P-value of Egger's test and the funnel plot.

## Results

### Literature selection

**Figure 1:**
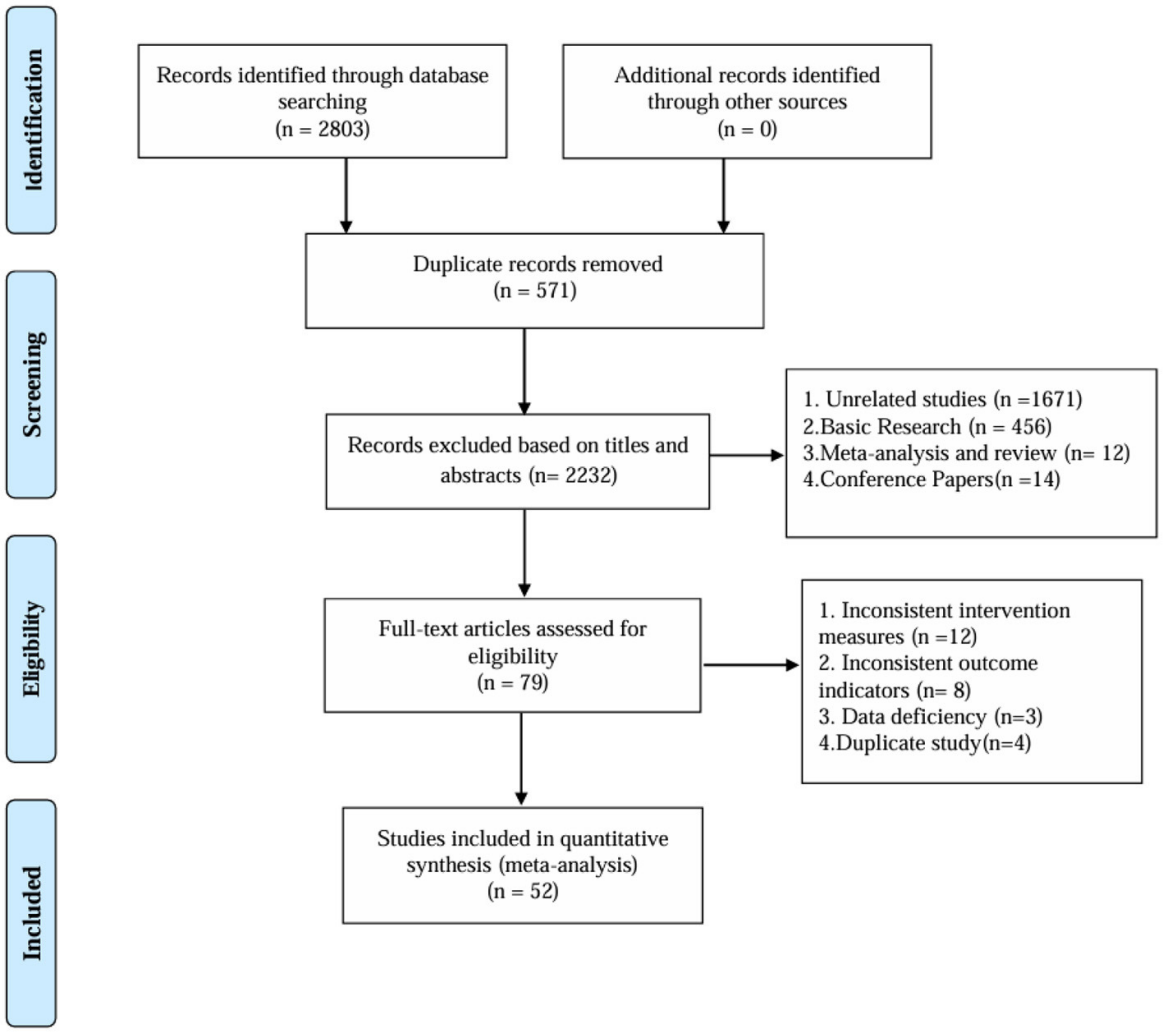
Flow chart.

After deleting duplicates, 2,803 records were retrieved, 571 duplicates were removed, 2,232 articles with inconsistent titles were deleted and 52 articles were finally included. The research flow chart is shown in Figure 1.

### Study and participant characteristics

**Figure 2:**
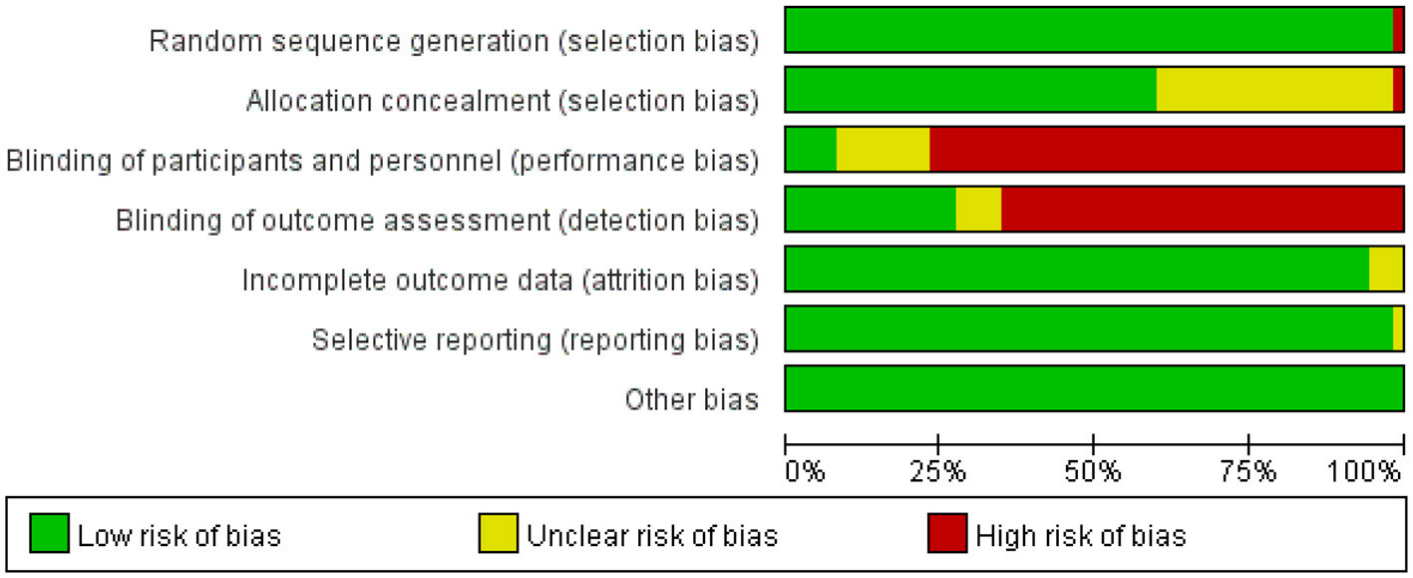
Percentage of studies examining the efficacy of exercise training in patients with non-specific chronic low back pain with low, unclear, and high risk of bias for each feature of the Cochrane Risk of Bias Tool.

The studies, published between 2009 and 2023, compared the effects of 25 different forms of exercise on quality of life in people with Parkinson's disease. The duration of intervention ranged from 3 weeks to 48 weeks. Of all the included studies, 45 reported quality of life, 14 reported depression, and 30 reported BBS. The characteristics of the studies and participants are shown in Table 2 and Appendix 3. The risk assessment of bias for each study is summarized in Appendix 4 and Figure 2.

**Table 2:** General characteristics of patients.

**Characteristics**	**Quality of life**	**Depression**	**Balance capacity**
***Publication characteristics***			
Total number of unique studies included	45	14	30
***Publication year***			
2001–2010	3	5	2
2011–2020	28	9	16
2021–2024	14	0	12
***Study design characteristics***			
***Range of study sample size***			
1–50	30	7	18
51–100	9	2	9
101–150	6	5	3
***No. of intervention arms included***			
2	43	14	2
3	2	0	28
***No. of studies containing the following treatment nodes***			
Aquatic Exercise (AQE)	3	0	1
Whole body vibration training (WBV)	1	0	1
Virtual reality (VR)	4	1	3
Treadmill training (TT)	1	0	0
Resistance training (RT)	4	1	2
Tai Chi (TC)	2	1	4
Biofeedback Balance and Gait Training (BGT)	0	0	1
Control group (CON)	10	3	5
Dance exercise (DE)	6	1	5
Balance training (BT)	3	1	1
Game training (GT)	2	0	0
Baduanjin (BDJ)	2	1	1
Home exercise (HE)	2	1	0
Yoga (YG)	0	1	0
Combined therapy (CT)	3	3	3
Traditional Rehabilitation (TR)	28	4	20
Stretch exercise (SE)	3	4	0
Five animal exercises (FAE)	6	3	3
Other exercise (OE)	5	2	4
Fitness exercise (FE)	2	0	2
Qigong (QG)	2	0	0
Virtual reality balance training (VRB)	1	0	1
Cycling exercise (CE)	2	0	1
Robotic Training (ROT)	0	0	1
Core strength training (CST)	1	0	1
**Time of intervention**			
During hospitalization	1	1	1
Unclear	1	0	1
3 weeks	1	0	0
4 weeks	2	0	2
5 weeks	4	1	4
6 weeks	6	2	3
8 weeks	12	4	8
10 weeks	2	0	2
11 weeks	1	0	0
12 weeks	6	2	2
16 weeks	1	0	1
20 weeks	1	0	1
24 weeks	6	4	4
48 weeks	1	0	1

### Quality of life

**Figure 3:**
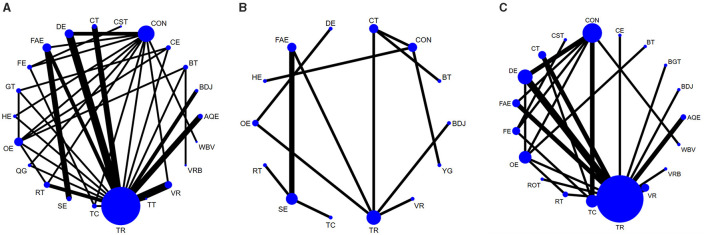
Network diagram of Quality of life **(A)**, Depression **(B)**, Balance capacity **(C)**. The node size represents the number of times the exercise appears in any comparison about that treatment, and the width of the edge represents the total sample size in the comparison it connects. AQE, Aquatic Exercise; WBV, Whole body vibration training; VR, Virtual reality; TT, Treadmill training; RT, Resistance training; TC, Tai Chi; BGT, Biofeedback Balance and Gait Training; CON, Control group; DE, Dance exercise; BT, Balance training; GT, Game training; BDJ, Baduanjin; HE, Home exercise; YG, Yoga; CT, Combined therapy; TR, Traditional Rehabilitation; SE, Stretch exercise; FAE, Five animal exercises; OE, Other exercise; FE, Fitness exercise; QG, Qigong; VRB, Virtual reality balance training; CE, Cycling exercise; ROT, Robotic Training; CST, Core strength training.

**Figure 4:**
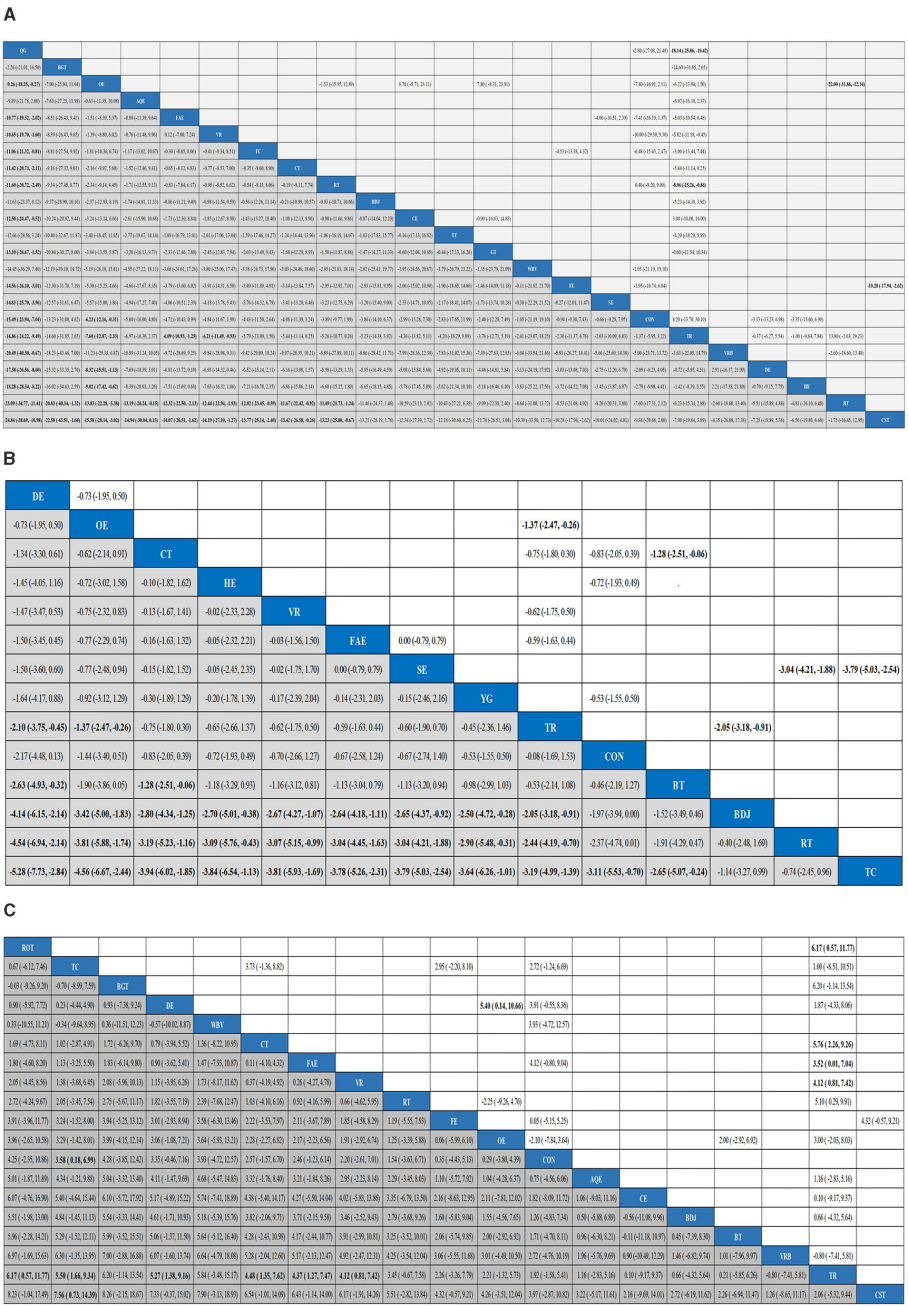
Results analysis league chart, Quality of life **(A)**, Depression **(B)**, Balance capacity **(C)**. The data are the mean difference and 95% confidence interval of continuous data. AQE, Aquatic Exercise; WBV, Whole body vibration training; VR, Virtual reality; TT, Treadmill training; RT, Resistance training; TC, Tai Chi; BGT, Biofeedback Balance and Gait Training; CON, Control group; DE, Dance exercise; BT, Balance training; GT, Game training; BDJ, Baduanjin; HE, Home exercise; YG, Yoga; CT, Combined therapy; TR, Traditional Rehabilitation; SE, Stretch exercise; FAE, Five animal exercises; OE, Other exercise; FE, Fitness exercise; QG, Qigong; VRB, Virtual reality balance training; CE, Cycling exercise; ROT, Robotic Training; CST, Core strength training.

A total of 45 studies evaluated Quality of life. We included the following 22 exercise measures in our network meta-analysis (Figure 3A): Aquatic Exercise (AQE), Whole body vibration training (WBV), Virtual reality (VR), Treadmill training (TT), Resistance training (RT), Tai Chi (TC), Control group (CON), Dance exercise (DE), Balance training (BT), Game training (GT), Baduanjin (BDJ), Home exercise (HE), Combined therapy (CT), Traditional Rehabilitation (TR), Stretch exercise (SE), Five animal exercises (FAE), Other exercise (OE), Fitness exercise (FE), Qigong (QG), Virtual reality balance training (VRB), Cycling exercise (CE), Core strength training (CST).Our results show that QG is higher than OE (MD, −9.26; 95%CI, −18.25 to −0.27), FAE (MD, −10.77; 95%CI −19.52 to −2.02), VR (MD, −10.65; 95%CI −19.70 to −1.60), TC (MD, −11.06; 95%CI −21.32 to −0.81), CT (MD, −11.42; 95%CI −20.73 to −2.11), RT (MD, −11.60; 95%CI −20.72 to −2.49), CE (MD −12.50; 95%CI −24.47 to −0.52), GT (MD −13.10; 95%CI −24.67 to −1.52), FE (MD −14.56; 95%CI −26.10 to −3.01), SE (MD −14.83; 95%CI −25.70 to −3.96), CON (MD −15.49; 95%CI −23.94 to −7.04), TR(MD −16.86; 95%CI −24.22 to −9.49), VRB(MD −20.49; 95%CI −40.30 to −0.67), DE(MD −17.58; 95%CI −26.56 to −8.60), HE(MD −18.28; 95%CI −28.34 to −8.22), BT(MD −23.09; 95%CI −34.77 to −11.41), CST(MD −24.84; 95%CI −38.69 to −10.98) to better improve quality of life in patients with Parkinson's disease (Figure 4A). In addition, we performed the Egger's test to assess publication bias (*P*= 0.352; Appendix 5.1). Heterogeneity and inconsistencies in the mesh meta-analysis were also evaluated (Appendix 6). We conducted a direct comparison of exercise interventions (Appendix 7.1).

### Depression

A total of 14 studies evaluated depression. We included the following 14 exercise measures in the network meta-analysis (Figure 3B): Virtual reality (VR), Resistance training (RT), Tai Chi (TC), Control group (CON), Dance exercise (DE), Balance training (BT), Baduanjin (BDJ), Home exercise (HE), Yoga (YG), Combined therapy (CT), Traditional Rehabilitation (TR), Stretch exercise (SE), Five animal exercises (FAE), Other exercise (OE). The results show that DE is superior to TR (SMD, −2.10; 95%CI −3.75 to −0.45), BT (SMD, −2.63; 95%CI −4.93 to −0.32), BDJ (SMD, −4.14; 95%CI −6.15 to −2.14), RT (SMD, −4.54; 95%CI −6.94 to −2.14), TC (SMD, −5.28; 95%CI −7.73 to −2.84) (Figure 4B). In addition, we performed the Egger test to assess publication bias (*P* = 0.952; Appendix 5.2). We also evaluated the heterogeneity and inconsistencies of the mesh meta-analysis (Appendix 6).

### Balance capacity

A total of 30 studies evaluated. We included the following 19 exercise measures in the network meta-analysis (Figure 3C): Aquatic Exercise (AQE), Whole body vibration training (WBV), Virtual reality (VR), Resistance training (RT), Tai Chi (TC), Biofeedback Balance and Gait Training (BGT), Control group (CON), Dance exercise (DE), Balance training (BT), Baduanjin (BDJ), Combined therapy (CT), Traditional Rehabilitation (TR), Five animal exercises (FAE), Other exercise (OE), Fitness exercise (FE), Virtual reality balance training (VRB), Cycling exercise (CE), Robotic Training (ROT), Core strength training (CST). The results show that: ROT is superior to TR (MD, 6.17; 95%CI 0.57 to 11.77) in improving balance capacity (Figure 4C). In addition, we assessed publication bias using the Egger test (*P* = 0.162; Appendix 5.3). We also evaluated heterogeneity and inconsistencies in the mesh meta-analysis (Appendix 6). We made a direct comparison of exercise interventions (Appendix 7.2).

## Discussion

Parkinson's disease is a chronic and progressive neurodegenerative disease, with a high incidence of non-motor symptoms such as insomnia, impulse control disorders, anxiety and depression (21), which seriously affect the quality of life of Parkinson's patients. Therefore, it is significant to identify exercise methods to improve the quality of life of PD patients. Our study included 52 studies exploring 25 exercise methods to clarify the best exercise to improve the quality of life in people with PD.

Our study found that QG is superior to OE, FAE, VR, TC, CT, RT, CE, GT, FE, SE, CON, TR, VRB, DE, HE, BT, and CST in improving the quality of life of PD patients. In our study, PDQ-39 is the evaluation index of PD patients' quality of life. In this study, PDQ-39 was used to evaluate patients' quality of life. Research has proved that PDQ-39 is a reliable and valid tool for assessing the functional impact of PD (22), demonstrating satisfactory internal consistency and stability (23). It offers clinicians a practical and reliable tool for efficiently assessing and standardizing the quality of life of patients with PD. In our study, QG was characterized as a low- to moderate-intensity aerobic exercise that integrates heart, breath, and body regulation. The exercise routine was carefully structured, with a moderate overall intensity. In practice, its focus on guiding qi, calming the mind, and promoting relaxation helps patients clear their thoughts and achieve a tranquil, relaxed state. The coordinated movements of qi and blood can channel and nourish the meridians, enhance circulation, strengthen muscles, and improve the physiological functions of various tissues and organs in the body (24).

QG can effectively enhance telomerase activity in patients with chronic fatigue by modulating the pituitary–thalamic–adrenal axis, reducing oxidative stress levels, and regulating immune responses. In addition, as a low-impact exercise, QG increases the concentration of serum insulin-like growth factor (IGF), which further promotes telomerase activity and helps delay cellular aging (25). Furthermore, studies have demonstrated that QG can enhance the regulatory mechanisms of the neural circuits connecting the cortex, pons, and cerebellum. This improvement contributes to greater precision in fine motor skills and, in turn, enhances the quality of life of patients with PD (26). Apathetic mood is a common symptom in patients with PD. QG, a low-intensity exercise, is well-suited to the physical and mental attributes of older adults. Communities should provide more accessible exercise spaces and enhance guidance on appropriate exercise methods for patients with PD to help improve their quality of life. Compared with VR, RT, and GT, QG is an effective and low-cost form of exercise. Moreover, long-term RT and VR can lead to increased muscle and visual fatigue in patients with PD. Studies have reported mild dizziness after VR, which ultimately increases discomfort for patients with PD (27). Many factors affect the quality of life of patients with PD. Studies have found that the age (28, 29), gender (29), duration of disease (30) and severity of disease (31, 32) course of PD patients can all affect the quality of life of PD patients. Therefore, the influence of other factors should be comprehensively considered when evaluating the impact of exercise interventions on the quality of life of patients with PD. However, our study observed that only QG was superior to other exercises in improving the quality of life of patients with PD. Notably, only two articles on QG were included in this meta-analysis.

Depression is a common negative emotion experienced by patients with PD (33). Depression is characterized by the loss of positive emotions and manifests as a reduced interest or pleasure in daily activities, inattention, and low mood (34). Repeated and persistent emotional stress can also contribute to the progression of neurodegenerative diseases. In our study, DE was more effective than TR, BT, BDJ, RT, and TC in improving depression in patients with PD. The American Dance Therapy Association (35) defines dance therapy as “a psychosomatic therapeutic exercise that promotes emotional, cognitive, physical, and social integration.” Dance therapy can improve social interaction among patients with PD, increase their enthusiasm for exercise, and effectively alleviate emotional disorders (35). Compared with BT and RT, dance is a highly social activity that offers opportunities for non-verbal communication and self-improvement (36, 37). Participating in group dance classes and dancing with partners provides valuable social interaction (38). Therefore, dance serves as movement therapy and improves social engagement, interpersonal relationships, and social support for patients with PD. According to the American Dance Therapy Association, dance therapy promotes emotional, cognitive, physical, and social integration. It helps patients with PD connect with others, improve interpersonal relationships, and prevent social isolation, which in turn eases depression. In addition, DE can reduce the progressive degeneration of neuronal axons, promote dendrite formation of new synapses, establish new neural connections, and activate or create new neural pathways. These improvements enhance the brain's regulatory control over the limbs, improve joint flexibility, and alleviate movement disorders (39). During dancing, external stimuli promote the release of various neurotransmitters, which help patients with PD train motor memory, improve balance and cognitive function, and alleviate depression (40).

However, considering that PD patients are usually elderly and have a stiff gait, they are particularly prone to fall when starting to run, turning around or avoiding obstacles. Therefore, special attention should be paid to the safety of the venue environment, and exercise should be carried out in open Spaces without obstacles as much as possible (41, 42). In addition, for PD patients, warm-up activities should be carried out before exercise to increase the movement of various body parts, posture coordination and breathing and prevent muscle strains (37). During the training process, family members of the patients should accompany and protect them and try to minimize unnecessary injuries. Considering that female PD patients are suspicious and sensitive, they are more likely to be unable to proceed smoothly due to the fear of falling. Psychological communication should be conducted when necessary to alleviate the patients' concerns.

Balance capacity refers to the ability to maintain the body's center of gravity within its base of support during both static (stationary) and dynamic (moving) tasks (43). Good balance control and quick postural adjustment to external disturbances are key factors for stable and efficient walking (44, 45). Our study found that ROT was superior to TR in improving balance in PD patients. ROT is based on the theory of central nervous system plasticity and functional reorganization, and a large number of repetitive and purposefully weight-loss walking training can also improve balance and contribute to the automation of gait and the improvement of pace (46, 47). The closed-chain movement assisted by the G-EOSystem^®^ lower limb rehabilitation robot increased the stimulation of the anti-gravity proprioception of the lower limb with decreased sensitivity. The central nervous system, supplemented by the external visual feedback system, mobilizes the body's postural regulation system and finally realizes the body's balance and stability by effectively exporting the coordinated contraction of the peripheral trunk and lower limb muscles (48). Furthermore, some studies have found that ROT can generate external rhythms through proprioceptive cue effects in a fixed pattern to compensate for the internal rhythm defects caused by abnormal basal ganglion circuits in patients with Parkinson's disease, which is helpful for maintaining normal gait (49, 50), thereby increasing the balance ability of PD patients.

### Strengths and limitations

First, this study is the most comprehensive and systematic comparative meta-analysis of the effects of exercise on quality of life in people with Parkinson's disease. In a reasonably large sample size of 52 studies, we included 25 exercise interventions. We compare them directly or indirectly with other interventions to provide new, comprehensive, evidence-based recommendations. In our research, we found that QG can effectively improve the quality of life of PD patients, and DE can improve the depression of PD patients. Overall, this study has specific clinical significance. Overall, our study also has certain limitations, and these results should be treated with caution as the sample size for each intervention is usually relatively small and has some influence on the results. Although we analyzed heterogeneity in the included literature, some factors between studies are unavoidable. The quality of life of PD patients is affected by many factors. We should also consider the influence of other factors when considering the quality of life and depression of PD patients. Unfortunately, different types of exercise set different exercise intensity standards, and many of the included studies did not report exercise intensity. Furthermore, it is important not to overlook the feasibility and accessibility of implementing the most effective physical intervention in clinical or community Settings. During the implementation of QG, DE, and ROT, attention should be paid to the compliance of PD patients, the required infrastructure, and professional training to ensure the best effect of the intervention. The prerequisite for PD patients is to ensure their safety. Therefore, there are requirements for aspects such as the venue, patient attire, and accompanying personnel. In future studies, researchers should report in detail the exercise intensity and exercise cycle and compare the impact of different exercise intensities and exercise cycles on the quality of life of PD patients to determine which exercise intensity is more conducive to improving the quality of life of PD patients.

## Conclusion

Our study found that QG improved the quality of life of people with Parkinson's disease better than other forms of exercise. DE can effectively improve depression in patients with Parkinson's disease, and ROT can effectively improve balance in patients with PD. In summary, exercise substantially affects PD's quality of life and negative emotions, but its internal molecular mechanism and the connection and regulation of neural circuits remain to be further explored. In addition, in the quality evaluation of this study, we found that many studies did not use blind methods and randomization, resulting in generally low evidential certainty of research results. Therefore, it is suggested that the survey quality be strictly controlled and the sample size be increased to verify this study's results further. Exercise has positive significance in the prevention, clinical practice and rehabilitation of PD. A standardized therapeutic effect evaluation system should be established for clinical medical staff to clarify the optimal training plan and parameter Settings for different disease courses and types of PD patients. Furthermore, due to the diversity of exercise methods, the focus of intervention for patients by various exercise methods is not the same, and thus the intervention effects are also different. PD patients should adopt a comprehensive intervention form that organically and scientifically combines multiple exercise methods to achieve the best intervention effect.

## Data Availability

The original contributions presented in the study are included in the article/Supplementary material, further inquiries can be directed to the corresponding author.
