# Supplementary material for: The effect of different exercise training modes on improving quality of life in patients with Parkinson's disease: a network analysis
Source: Front Neurol. 2025 Jul 2;16:1601080. doi: 10.3389/fneur.2025.1601080 (PMC12264356; doi:10.3389/fneur.2025.1601080)
Supplement: Supplementary file 1 [file Data_Sheet_1.zip › Supplementary Material/Appendix 1-Search strategy.DOCX]

**Pubmed:**

| Number | Search terms | Results |
| --- | --- | --- |
| **#1** | ((((((((((Parkinson Disease[MeSH Terms]) OR (Idiopathic Parkinson's Disease[Title/Abstract])) OR (Lewy Body Parkinson's Disease[Title/Abstract])) OR (Parkinson's Disease, Idiopathic[Title/Abstract])) OR (Parkinson's Disease, Lewy Body[Title/Abstract])) OR (Paralysis Agitans[Title/Abstract])) OR (Parkinson's Disease[Title/Abstract])) OR (Idiopathic Parkinson Disease[Title/Abstract])) OR (Lewy Body Parkinson Disease[Title/Abstract])) OR (Primary Parkinsonism Parkinsonism, Primary[Title/Abstract])) OR (Parkinson Disease, Idiopathic[Title/Abstract]) | 140,932 |
| **#2** | ((((((((((((((((((((((((((((((((((((Exercises[Title/Abstract]) OR (Exercise, Physical[Title/Abstract])) OR (Exercises, Physical[Title/Abstract])) OR (Physical Exercise[Title/Abstract])) OR (Physical Exercises[Title/Abstract])) OR (Physical Activity[Title/Abstract])) OR (Activities, Physical[Title/Abstract])) OR (Activity, Physical[Title/Abstract])) OR (Physical Activities[Title/Abstract])) OR (Exercise, Aerobic[Title/Abstract])) OR (Aerobic Exercise[Title/Abstract])) OR (Aerobic Exercises[[Title/Abstract])) OR (Exercises, Aerobic[Title/Abstract])) OR (Exercise, Isometric[Title/Abstract])) OR (Exercises, Isometric[Title/Abstract])) OR (Isometric Exercises[Title/Abstract])) OR (Isometric Exercise[Title/Abstract])) OR (Aquatic Exercise[Title/Abstract])) OR (Whole body vibration training[Title/Abstract])) OR (Virtual reality[Title/Abstract])) OR (Treadmill training[Title/Abstract])) OR (Resistance training[Title/Abstract])) OR (Tai Chi[Title/Abstract])) OR (Biofeedback Balance[Title/Abstract] AND Gait Training[Title/Abstract])) OR (Dance exercise[Title/Abstract])) OR (Balance training (BT[Title/Abstract]))) OR (Game training (GT[Title/Abstract]))) OR (Baduanjin (BDJ[Title/Abstract]))) OR (Home exercise (HE[Title/Abstract]))) OR (Yoga[Title/Abstract])) OR (Combined therapy[Title/Abstract])) OR (Stretch exercise[Title/Abstract])) OR (Five animal exercises[Title/Abstract])) OR (Fitness exercise[Title/Abstract])) OR (Qigong[Title/Abstract])) OR (Cycling exercise[Title/Abstract])) OR (Robotic Training[Title/Abstract]) | 313,036 |
| **#3** | #1 AND #2 | 2472 |
| **#4** | ((((Quality of life[MeSH Terms]) OR (Life Quality[Title/Abstract])) OR (Health-Related Quality Of Life[Title/Abstract])) OR (Health Related Quality Of Life[Title/Abstract])) OR (HRQOL[Title/Abstract]) | 9681 |
| **#5** | #3 AND #4 | 380 |
