# Supplementary material for: The effect of different exercise training modes on improving quality of life in patients with Parkinson's disease: a network analysis
Source: Front Neurol. 2025 Jul 2;16:1601080. doi: 10.3389/fneur.2025.1601080 (PMC12264356; doi:10.3389/fneur.2025.1601080)
Supplement: Supplementary file 1 [file Data_Sheet_1.zip › Supplementary Material/Appendix 3-Included literature.docx]

***General characteristics of patients***

| **Author** | **Publish**  **Year** | **Country** | **measure** | **Age**  **(Mean ± SD)** | **Number**  **(male/** **female)** | **duration of diagnosis**  **(year/month)** | **Intervention**  **time** | **Intervention**  **frequency** | **Outcomes** |
| --- | --- | --- | --- | --- | --- | --- | --- | --- | --- |
| Cheng^[1]^ | 2019 | China | VR/TR | 59.2±7.3/  58.6±7.5 | 17/23 | 6.1±1.4 /  6.2±1.7 | 8weeks | 2 times a week | ①③ |
| Cao^[2]^ | 2021 | China | FAE/TR | 62.45±5.13/  66.48±5.15 | 41/21 | 6.10±0.87/  5.71±1.10 | 8 weeks | Once a week | ①③ |
| Shen^[3]^ | 2022 | China | CT/TR | 66.48±5.15 | 35/27 | NA | 24 weeks | 2 times a week | ①③ |
| Volpe^[4]^ | 2016 | Italy | AQE/TR | 70.6±7.8/  70±7.8 | 19/11 | 9.4±7.5/  9±7.0 | 8 weeks | NA | ①③ |
| Picelli^[5]^ | 2012 | Italy | ROT/TR | 68.3 | 20/14 | 7.5±3.45 | 4 weeks | 3 days a week | ③ |
| Michels^[6]^ | 2018 | America | DT/CONP | 66.44/75.50 | NA | 3.76±2.88/  5.94±3.61 | 12 weeks | NA | ①③ |
| Kurt^[7]^ | 2017 | Turkey | TC/TR | 62.41±6.76/  63.61±7.18 | 24/16 | NA | 5 weeks | 5 times a week | ①③ |
| Volpe^[8]^ | 2013 | Italy | DT/TR | 61.6±4.5/  65.0±5.3 | 13/11 | 9.0±3.6/  8.9±2.5 | 24 weeks | Once a week | ①③ |
| Ventura^[9]^ | 2016 | America | DT/CON | 71.8±3.6/  70.4±5.5 | 2/13 | 6.1±3.1/  4.3±2.6 | 10 weeks | Once a week | ①③ |
| Yang^[10]^ | 2016 | China | VR/BT | 72.5±8.4 75.4±6.3 | 9/14 | 9.4±3.6/  8.3±4.1 | 6 weeks | 2 times a week | ①③ |
| Carpinella^[11]^ | 2017 | Italy | BGT/TR | 73.0±7.1/  75.6±8.2 | 23/14 | 7.5±3.2/  10.3±5.7 | 20 weeks | 3 times a week | ①③ |
| Volpe^[12]^ | 2014 | Italy | AQE/TR | 68±7/  66±8 | NA | 7.5±5.1/  7.6±4.63 | 8 weeks | 5 times a week | ①③ |
| Qutubuddin^[13]^ | 2012 | America | CE/TR | NA | 13/10 | NA | 8 weeks | 2 times a week | ①③ |
| Gao^[14]^ | 2022 | China | TC/FE/CON | 64±5/  65±8/  63±6 | 24/39 | NA | 16 weeks | 3 times a week | ①③ |
| Zhu^[15]^ | 2022 | China | RT/TR | 67.8±4.6/  69.3±5.1 | 46/32 | 3.7±1.6/  3.3±1.4 | 6 weeks | 5 times a week | ②③ |
| Li^[16]^ | 2021 | China | VR/TR | 57.33±8.73/ 57.04±7.15 | 28/20 | NA | 8 weeks | 3 times a week | ①②③ |
| Liu^[17]^ | 2023 | China | BDJ/TR | 63.69±5.04/  64.05±4.54 | 82/34 | NA | During hospitalization | NA | ①③ |
| Zhang^[18]^ | 2022 | China | FAE/TR | 67.37±6.53 | 68/52 | 8.12±6.15 | 6 weeks | NA | ①②③ |
| Han^[19]^ | 2021 | China | CT/TR | 68.60±7.37  67.88±8.63 | 49/51 | 36(24, 120)/  36(24, 111) M | 8 weeks | 3 times a week | ①②③ |
| You^[20]^ | 2020 | China | TC/CT | 68.81±5.02/ 68.49±5.27 | 37/33 | 4.21±0.24/  4.17±0.35 | 24 weeks | 2 times a week | ②③ |
| Zhang^[21]^ | 2019 | China | FAE/CON | 65.06±4.905/ 63.94±5.068 | 13/21 | 2.59±1.58/  2.47±1.12 | 48 weeks | 2 times a week | ①③ |
| Xi^[22]^ | 2022 | China | OE/TR | 67.41±4.42/  67.02±4.52 | 33/33 | 4.00±2.06/  3.36±1.52 | 8 weeks | 5 times a week | ①②③ |
| Wang^[23]^ | 2023 | China | TC/CON | 72.07±8.33/  67.13±8.33 | 18/12 | 4.27±3.31/  5.87±2.72 | 24 weeks | 3 times a week | ②③ |
| Wang^[24]^ | 2022 | China | FAE/CST | 63.24±4.53/ 63.14±4.58 | 50/60 | 5.25±0.74/  5.35±0.82 | NA | NA | ①③ |
| Kunkel^[25]^ | 2017 | UK | DT/TR | 71.3±7.7/ 69.7±6.0 | 25/26 | 4.7±3.5/  7.0±4.9 | 10 weeks | 2 times a week | ①③ |
| Arias^[26]^ | 2009 | Spain | WBV/CON | 66.90±11.11/ 66.55±5.57 | NA | NA | 5 weeks | NA | ①③ |
| van^[27]^ | 2014 | Netherlands | OE/BT | 20/13 | 68.8±6.39/  68.8±9.68 | 9.0/8.8 | 5 weeks | 2 times a week | ①②③ |
| Dibble^[28]^ | 2009 | USA | RT/OE | NA | 64.3±9.6/  67.0±10.2 | 6.1±3.9/  6.5±4.3 | 5 weeks | 3 times a week | ①③ |
| Hashimoto^[29]^ | 2015 | Japan | DE/OE/CON | 67.9±7.0/  62.7±14.9 | 12/34 | 6.3±4.6/  7.8±6.2/  6.9±4.0 | 12 weeks | Once a week | ②③ |
| Santos^[30]^ | 2017 | Spain | RT/TR | 73.38±8.81/  73.80±7.05 | 15/13 | 10.84±4.09/  10.46±4.01 | 8 weeks | 2 times a week | ① |
| Rios^[31]^ | 2015 | Canada | DE/CON | 63.2±9.9/ 64.3±8.1 | 19/14 | 5.5±4.4/  7.7±4.6 | 12 weeks | NA | ① |
| Tollár^[32]^ | 2018 | Hungary | OE/CON | 67.3±3.4、67.6±4.1 | 29/26 | NA | 3 weeks | 5 times a week | ① |
| Allen^[33]^ | 2010 | Australia | BT/TR | 66±10/  68±7 | 26/22 | 7±5/  9±6 | 24 weeks | 3 times a week | ① |
| Carroll^[34]^ | 2017 | Ireland | AQE/TR | 69.5/74 | 12/6 | NA | 6 weeks | 2 times a week | ① |
| McKee^[35]^ | 2013 | America | DE/CON | 68.4±7.5/ 74.4±6.5 | 20/13 | 7.0±5.5/  7.2±4.9 | 4 weeks | NA | ①③ |
| Moon^[36]^ | 2020 | America | QG/CON | 66.4±8.1 65.9±5.4 | 10/7 | 4.25±2.1/  5.33±3.3 | 12 weeks | 2 times a week | ① |
| Morris^[37]^ | 2015 | Australia | RT/CON | 67.4±10.4/  67.9±8.4 | 140/70 | 7.2±6.2/  6.9±5.2 | 8 weeks | Once a week | ① |
| Canning^[38]^ | 2022 | Australia | TT/TR | 60.7±5.9/ 62.9±9.9 | 11/9 | 6.1±4.0/  5.2 ±4.1 | 6 weeks | 4 times a week | ① |
| Liao^[39]^ | 2014 | China | VR/TR/CON | 67.3±7.1/ 65.1±6.7/ 64.6±8.6 | 17/19 | 7.9±2.7/  6.9±2.8/  6.4±3.0 | 6 weeks | 2 times a week | ① |
| Ferraz^[40]^ | 2018 | Brazil | OE/CE/GT | NA | 37/25 | NA | 8 weeks | NA | ① |
| Glicia^[41]^ | 2013 | Brazil | VR/TR | NA | 22/19 | NA | 4 weeks | 3 times a week | ① |
| Morris^[42]^ | 2017 | Australia | HE/TR | 71±8 /  71±10 | 80/53 | NA | 6 weeks | 2 times a week | ① |
| Allen ^[43]^ | 2017 | Australia | GT/TR | 67.5±7.3/  68.4±8.5 | 33/5 | 7.9±3.9/  8.7±6.1 | 12 weeks | 3 times a week | ① |
| Ferreira^[44]^ | 2018 | Brasil | RT/TR | 64.1±7. 0/  67.6±8.9 | NA | 6.4±2.7/  4.5±4.0 T: | 24 weeks | 2 times a week | ① |
| Li^[45]^ | 2021 | China | FAE/SE | 67.57±3.95/  70±5.59 | 29/11 | 6.83±4.09/  7.76±4.55 | 12 weeks | 2 times a week | ① |
| Chang^[46]^ | 2020 | China | CT/TR | 69±2/  67±11 | 76/44 | NA | 11 weeks | Once a day | ① |
| Wang^[47]^ | 2023 | China | FAE/SE | 68.96±4.30 /  68.29±4.86 | 23/25 | 6.56±3.91/  6.22±3.66 | 24 weeks | 2 times a week | ①② |
| He^[48]^ | 2022 | China | FAE/SE | 67.76±4.34/  68.42±5.34 | 25/26 | 5.60±3.39/  5.88±4.16 | 24 weeks | 2 times a week | ①② |
| Li^[49]^ | 2019 | China | TR/BDJ | 62.88±7.50/  62.42±9.37 | 39/27 | NA | During hospitalization | NA | ② |
| Wu^[50]^ | 2023 | China | HE/CON | 71.93±5.85/  73.81±6.2 | 16/15 | 6.9[4.8,8.9]/  3.95[2.7,9.37] | 12 weeks | 5 times a week | ①② |
| Jiang^[51]^ | 2023 | China | QG/TR | 60.60±6.75/  59.45±5.45 | 20/20 | 4.30±1.38/  3.85±1.35 | 8 weeks | 5 times a week | ① |
| Kwok^[52]^ | 2019 | China | YG/TR | 63.7±8.2/  63.5±9.3 | 65/73 | NA | 8 weeks | NA | ② |

①PDQ39 ②HAMD ③BBS

[1] YUANYUANA C, YANGA Y, SIQUANB L, et al. Effect of Virtual Ｒeality Technology Combined with Ｒehabilitation Training on Balance Function of Patients with Parkinson's Disease Medical Ｒecapitulate. 2019;25(21):4325-4329.

[2] HAI-HAO C, WEN-YU S, XIAO-MING X, et al. Effects of Wuqinxi on Balance, Walking and Quality of Life for Patients with Parkinson's Disease. Chin J Rehabil Theory Pract. 2021;27(09):1087-1092.

[3] BIN S, XIANG Q, YAN S, et al. Effects of vestibular rehabilitation training combined with gait training on Webster score, BBS score and fall incidence in elderly patients with Parkinson's disease. Chinese Journal of Gerontology. 2022;42(03):614-617.

[4] VOLPE D, GIANTIN MG, MANUELA P, et al. Water-based vs. non-water-based physiotherapy for rehabilitation of postural deformities in Parkinson's disease: a randomized controlled pilot study. Clin Rehabil. 2017;31(8):1107-1115.

[5] PICELLI A, MELOTTI C, ORIGANO F, et al. Does robotic gait training improve balance in Parkinson's disease? A randomized controlled trial. Parkinsonism Relat Disord. 2012;18(8):990-993.

[6] MICHELS K, DUBAZ O, HORNTHAL E, et al. "Dance Therapy" as a psychotherapeutic movement intervention in Parkinson's disease. Complement Ther Med. 2018;40:248-252.

[7] KURT EE, BüYüKTURAN B, BüYüKTURAN Ö, et al. Effects of Ai Chi on balance, quality of life, functional mobility, and motor impairment in patients with Parkinson's disease<sup/>. Disabil Rehabil. 2018;40(7):791-797.

[8] VOLPE D, SIGNORINI M, MARCHETTO A, et al. A comparison of Irish set dancing and exercises for people with Parkinson's disease: a phase II feasibility study. BMC Geriatr. 2013;13:54.

[9] VENTURA MI, BARNES DE, ROSS JM, et al. A pilot study to evaluate multi-dimensional effects of dance for people with Parkinson's disease. Contemp Clin Trials. 2016;51:50-55.

[10] YANG WC, WANG HK, WU RM, et al. Home-based virtual reality balance training and conventional balance training in Parkinson's disease: A randomized controlled trial. J Formos Med Assoc. 2016;115(9):734-743.

[11] CARPINELLA I, CATTANEO D, BONORA G, et al. Wearable Sensor-Based Biofeedback Training for Balance and Gait in Parkinson Disease: A Pilot Randomized Controlled Trial. Arch Phys Med Rehabil. 2017;98(4):622-630.e623.

[12] VOLPE D, GIANTIN MG, MAESTRI R, et al. Comparing the effects of hydrotherapy and land-based therapy on balance in patients with Parkinson's disease: a randomized controlled pilot study. Clin Rehabil. 2014;28(12):1210-1217.

[13] QUTUBUDDIN A, REIS T, ALRAMADHANI R, et al. Parkinson's disease and forced exercise: a preliminary study. Rehabil Res Pract. 2013;2013:375267.

[14] SUN G. Research on the Effects of Tai Chi on Motor Symptoms of Patients with Parkinson’s Disease and Its Promotion Based on the Internet Platform in Song Gao. 2022.

[15] TONG Z, JIUYING S, CAIHUA S. Application of Ｒesistance Exercise Combined with Ｒegular Ｒehabilitation Training in the Nursing of Patients with Parkinson' s Disease. Qilu Nursing Journal. 2022;28(05):22-25.

[16] RUNZE L. An Application Study of Evidence-Based Virtual Reality Rehabilitation Training for Patients with Parkinson's Disease. 2021.

[17] FENGCHUN L, QIAN M, XIAOMENG S, et al. The application of Baduanjin exercise in patients with Parkinson's disease based on the theory of interactive standard. CHINESE NURSING RESEARCH. 2023;37(18):3388-3391.

[18] GUOJUAN Z, HONGXIA Z, JUAN L, et al. Application of Wuqinxi combined with physical training in patients with Parkinson's disease. Chinese Clinical Nursing. 2022;14(05):287-290.

[19] WEN H, TING D, XINYUAN D, et al. Application of motion-sensing games combined with five-fowl play in patients with motor disorders of Parkinson's disease. GuangxiMedical Journal. 2021;43(09):1153-1156+1160.

[20] YE Y, JUNHUI S. Effect of Tai Chi balance exercise group therapy on improving balance function and depression in patients with Parkinson's disease. Guizhou Medical Journal. 2020;44(07):1071-1072.

[21] GAI Z. Research on the Intervention Effect of Health Qigong Wuqinxi on theWalking and Balance Ability of Patients with Parkinson's Disease. 2019.

[22] XIAOMING X, BIHONGYAN. Effect of abdominaltorsion movement on depression,constipation,motorsymptomsand quality of life in patient swith Parkinson's disease. ChinJ Rehabil Theory Pract. 2022;28(02):220-226.

[23] MEIHUA W, MIN G, HAIQIN W, et al. Rehabilitation effect of Taijiquan with different training loads on patients with early and middle Parkinson's disease. Progress in Biochemistry and Biophysics. 2023;50(10):2487-2495.

[24] RAN W, HUIZI W, QINGJUAN W, et al. Intervention eff ect of bench exercise on gait, balance ability and quality of life of patients with Parkinson dyskinesia. Nursing Practice and Research. 2022;19(01):66-69.

[25] KUNKEL D, FITTON C, ROBERTS L, et al. A randomized controlled feasibility trial exploring partnered ballroom dancing for people with Parkinson's disease. Clin Rehabil. 2017;31(10):1340-1350.

[26] DINCHER A, BECKER P, WYDRA G. Effect of whole-body vibration on freezing and flexibility in Parkinson's disease-a pilot study. Neurol Sci. 2021;42(7):2795-2801.

[27] VAN DEN HEUVEL MR, KWAKKEL G, BEEK PJ, et al. Effects of augmented visual feedback during balance training in Parkinson's disease: a pilot randomized clinical trial. Parkinsonism Relat Disord. 2014;20(12):1352-1358.

[28] DIBBLE LE, HALE TF, MARCUS RL, et al. High intensity eccentric resistance training decreases bradykinesia and improves Quality Of Life in persons with Parkinson's disease: a preliminary study. Parkinsonism Relat Disord. 2009;15(10):752-757.

[29] HASHIMOTO H, TAKABATAKE S, MIYAGUCHI H, et al. Effects of dance on motor functions, cognitive functions, and mental symptoms of Parkinson's disease: a quasi-randomized pilot trial. Complement Ther Med. 2015;23(2):210-219.

[30] SANTOS L, FERNANDEZ-RIO J, WINGE K, et al. Effects of progressive resistance exercise in akinetic-rigid Parkinson's disease patients: a randomized controlled trial. Eur J Phys Rehabil Med. 2017;53(5):651-663.

[31] RIOS ROMENETS S, ANANG J, FERESHTEHNEJAD SM, et al. Tango for treatment of motor and non-motor manifestations in Parkinson's disease: a randomized control study. Complement Ther Med. 2015;23(2):175-184.

[32] TOLLáR J, NAGY F, KOVáCS N, et al. A High-Intensity Multicomponent Agility Intervention Improves Parkinson Patients' Clinical and Motor Symptoms. Arch Phys Med Rehabil. 2018;99(12):2478-2484.e2471.

[33] ALLEN NE, CANNING CG, SHERRINGTON C, et al. The effects of an exercise program on fall risk factors in people with Parkinson's disease: a randomized controlled trial. Mov Disord. 2010;25(9):1217-1225.

[34] CARROLL LM, VOLPE D, MORRIS ME, et al. Aquatic Exercise Therapy for People With Parkinson Disease: A Randomized Controlled Trial. Arch Phys Med Rehabil. 2017;98(4):631-638.

[35] MCKEE KE, HACKNEY ME. The effects of adapted tango on spatial cognition and disease severity in Parkinson's disease. J Mot Behav. 2013;45(6):519-529.

[36] MOON S, SARMENTO CVM, STEINBACHER M, et al. Can Qigong improve non-motor symptoms in people with Parkinson's disease - A pilot randomized controlled trial? Complement Ther Clin Pract. 2020;39:101169.

[37] MORRIS ME, MENZ HB, MCGINLEY JL, et al. A Randomized Controlled Trial to Reduce Falls in People With Parkinson's Disease. Neurorehabil Neural Repair. 2015;29(8):777-785.

[38] CANNING CG, ALLEN NE, DEAN CM, et al. Home-based treadmill training for individuals with Parkinson's disease: a randomized controlled pilot trial. Clin Rehabil. 2012;26(9):817-826.

[39] LIAO YY, YANG YR, CHENG SJ, et al. Virtual Reality-Based Training to Improve Obstacle-Crossing Performance and Dynamic Balance in Patients With Parkinson's Disease. Neurorehabil Neural Repair. 2015;29(7):658-667.

[40] FERRAZ DD, TRIPPO KV, DUARTE GP, et al. The Effects of Functional Training, Bicycle Exercise, and Exergaming on Walking Capacity of Elderly Patients With Parkinson Disease: A Pilot Randomized Controlled Single-blinded Trial. Arch Phys Med Rehabil. 2018;99(5):826-833.

[41] PEDREIRA G, PRAZERES A, CRUZ D, et al. Virtual games and quality of life in Parkinson’s disease: A randomised controlled trial 2013:Vol.2, No.4, 97-101

[42] MORRIS ME, TAYLOR NF, WATTS JJ, et al. A home program of strength training, movement strategy training and education did not prevent falls in people with Parkinson's disease: a randomised trial. J Physiother. 2017;63(2):94-100.

[43] ALLEN NE, SONG J, PAUL SS, et al. An interactive videogame for arm and hand exercise in people with Parkinson's disease: A randomized controlled trial. Parkinsonism Relat Disord. 2017;41:66-72.

[44] FERREIRA RM, ALVES W, DE LIMA TA, et al. The effect of resistance training on the anxiety symptoms and quality of life in elderly people with Parkinson's disease: a randomized controlled trial. Arq Neuropsiquiatr. 2018;76(8):499-506.

[45] ZHENLAN L. Effects of Exercise Intervention on Gait during Different Tasks for Patients with Mild to Moderate Parkinson's disease. 2021.

[46] ZIHAN C. Inflammation and oxidative stress in PD and evaluation theefficacy of intensive training & virtual reality rehabilitationon gait disorder and motor function in PD patients. 2020.

[47] ZHEN W. Electrophysiological characteristics of motor inhibition in Parkinson's disease and the empirical study of exercise intervention 2023.

[48] JIE H. Effects of 12 Week Wuqinxi Intervention on Executive Function in Patients with Parkinson's Disease. 2022.

[49] CUIJING L, HONG C, NA W, et al. Effect of Baduanjin exercise therapy on quality of life and depressive symptoms in patients with Parkinson's disease complicated with mild to moderate depression. Beijing Journal of Traditional Chinese Medicine. 2019;38(11):1129-1131.

[50] YUXIA W. The intervention study of home-based rehabilitation exerciseon motor function and quality of life in elderly patients withParkinson's disease. 2023.

[51] JIAHUI J, HONGYAN B. Influence of fitness qigong Yijinjing on fatigue and quality of life in patients with Parkinson's disease. CHINESE NURSING RESEARCH 2023;37(24):4452-4457.

[52] KWOK JYY, KWAN JCY, AUYEUNG M, et al. Effects of Mindfulness Yoga vs Stretching and Resistance Training Exercises on Anxiety and Depression for People With Parkinson Disease: A Randomized Clinical Trial. JAMA Neurol. 2019;76(7):755-763.
