# Supplementary material for: The effect of different exercise training modes on improving quality of life in patients with Parkinson's disease: a network analysis
Source: Front Neurol. 2025 Jul 2;16:1601080. doi: 10.3389/fneur.2025.1601080 (PMC12264356; doi:10.3389/fneur.2025.1601080)
Supplement: Supplementary file 1 [file Data_Sheet_1.zip › Supplementary Material/Appendix 4-Risk of bias summary.pdf]

	Random sequence generation (selection bias)	Allocation concealment (selection bias)	Blinding of participants and personnel (performance bias)	Binding of outcome assessment (detection bias)	Incomplete outcome data (attrition bias)	Selective reporting (reporting bias)	Other bias
Allen 2010	+	?	?	−	+	+	+
Allen 2015	+	+	−	+	+	+	+
Arias 2009	+	+	+	+	+	+	+
Canning 2022	+	+	+	+	+	+	+
Cao 2021	+	?	−	−	+	+	+
Carpinella 2017	+	?	−	−	+	+	+
Carroll 2017	+	+	+	+	+	+	+
Chang 2020	+	?	−	−	+	+	+
Cheng 2019	+	+	−	−	?	+	+
Dibble 2009	+	?	−	−	+	+	+
Ferraz 2018	+	+	−	+	+	+	+
Ferreira 2018	+	+	?	+	+	+	+
Gao 2022	+	?	−	−	+	+	+
Glicia 2013	+	?	−	−	+	+	+
Han 2021	+	?	−	−	+	+	+
Hashimoto 2015	+	+	−	−	+	+	+
He 2022	+	?	−	−	+	+	+
Jiang 2023	+	+	−	−	+	+	+
Kunkel 2017	+	+	+	+	+	+	+
Kurt 2017	+	+	−	−	+	+	+
Kwok 2019	+	+	−	−	+	+	+
Li 2019	+	+	−	−	+	+	+
Li 2021	+	+	−	−	+	+	+
Li 2021	+	+	?	+	+	+	+
Liao 2014	+	+	−	−	?	?	+
Liu 2017	+	?	−	−	+	+	+
McKee 2013	+	+	−	+	+	+	+
Michels 2018	+	+	?	?	+	+	+
Moon 2020	+	?	−	−	+	+	+
Morris 2015	+	+	?	?	+	+	+
Morris 2017	+	+	?	+	+	+	+
Picelli 2012	+	?	−	−	+	+	+
Qutubuddin 2012	+	+	−	+	+	+	+
Rios 2015	+	+	?	?	?	+	+
Santos 2017	+	+	?	?	+	+	+
Shen 2022	+	?	−	−	+	+	+
Tollár 2018	+	?	−	−	+	+	+
van 2014	+	+	−	−	+	+	+
Ventura 2016	−	−	−	−	+	+	+
Volpe 2013	+	+	−	+	+	+	+
Volpe 2014	+	+	−	+	+	+	+
Volpe 2016	+	+	−	+	+	+	+
Wang 2017	+	?	−	−	+	+	+
Wang 2022	+	?	−	−	+	+	+
Wang 2023	+	+	−	−	+	+	+
Wu 2023	+	+	−	−	+	+	+
Xi 2022	+	+	−	−	+	+	+
Yang 2016	+	+	−	−	+	+	+
You 2020	+	?	−	−	+	+	+
Zhang 2019	+	?	−	−	+	+	+
Zhang 2022	+	?	−	−	+	+	+
Zhu 2022	+	?	−	−	+	+	+
