# Supplementary figures and images for: The effect of different exercise training modes on improving quality of life in patients with Parkinson's disease: a network analysis
Source: Front Neurol. 2025 Jul 2;16:1601080. doi: 10.3389/fneur.2025.1601080 (PMC12264356; doi:10.3389/fneur.2025.1601080)

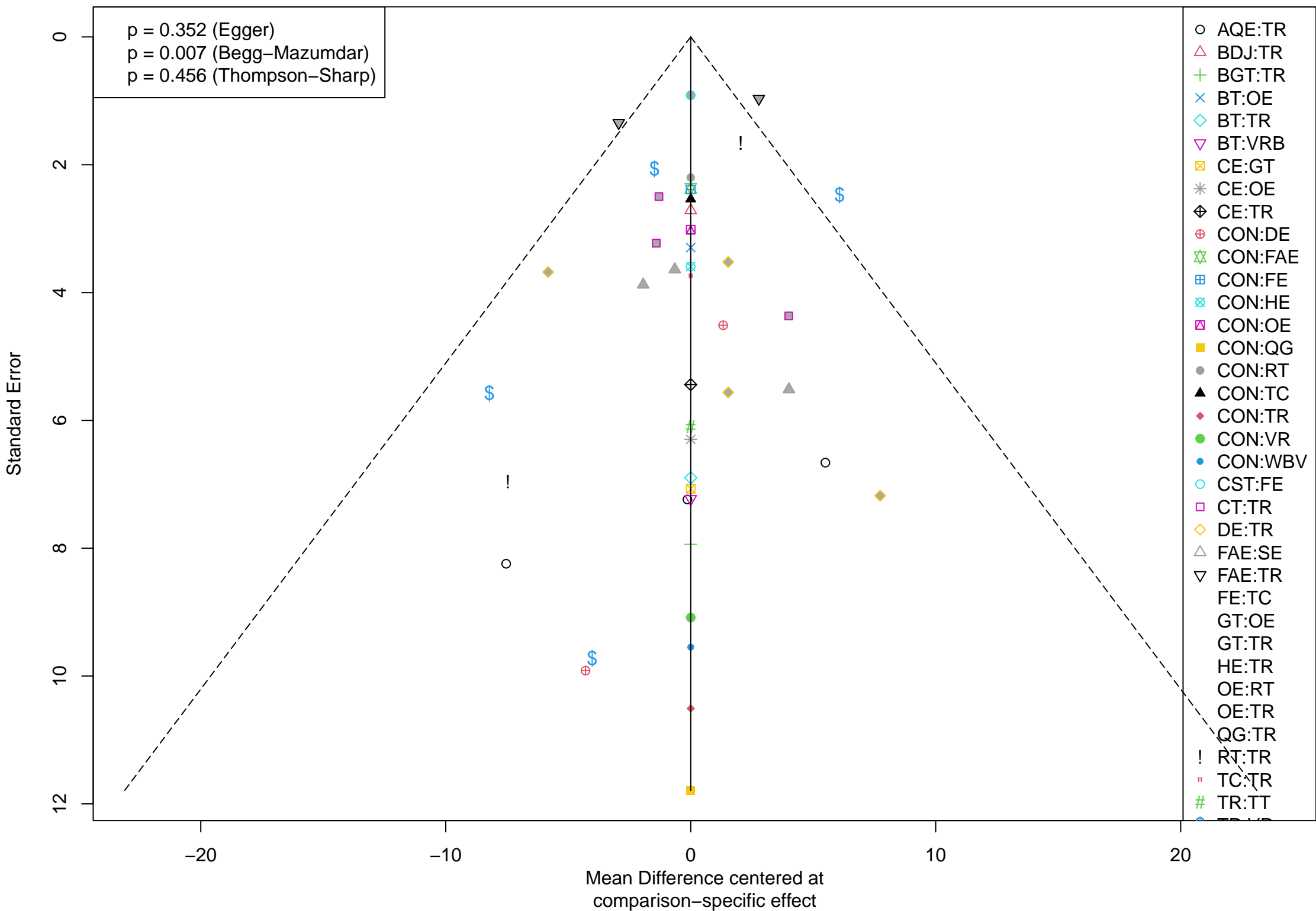

Supplement: Supplementary file 1 [file Data_Sheet_1.zip › Supplementary Material/Appendix 5.1-Funnel polt.pdf]

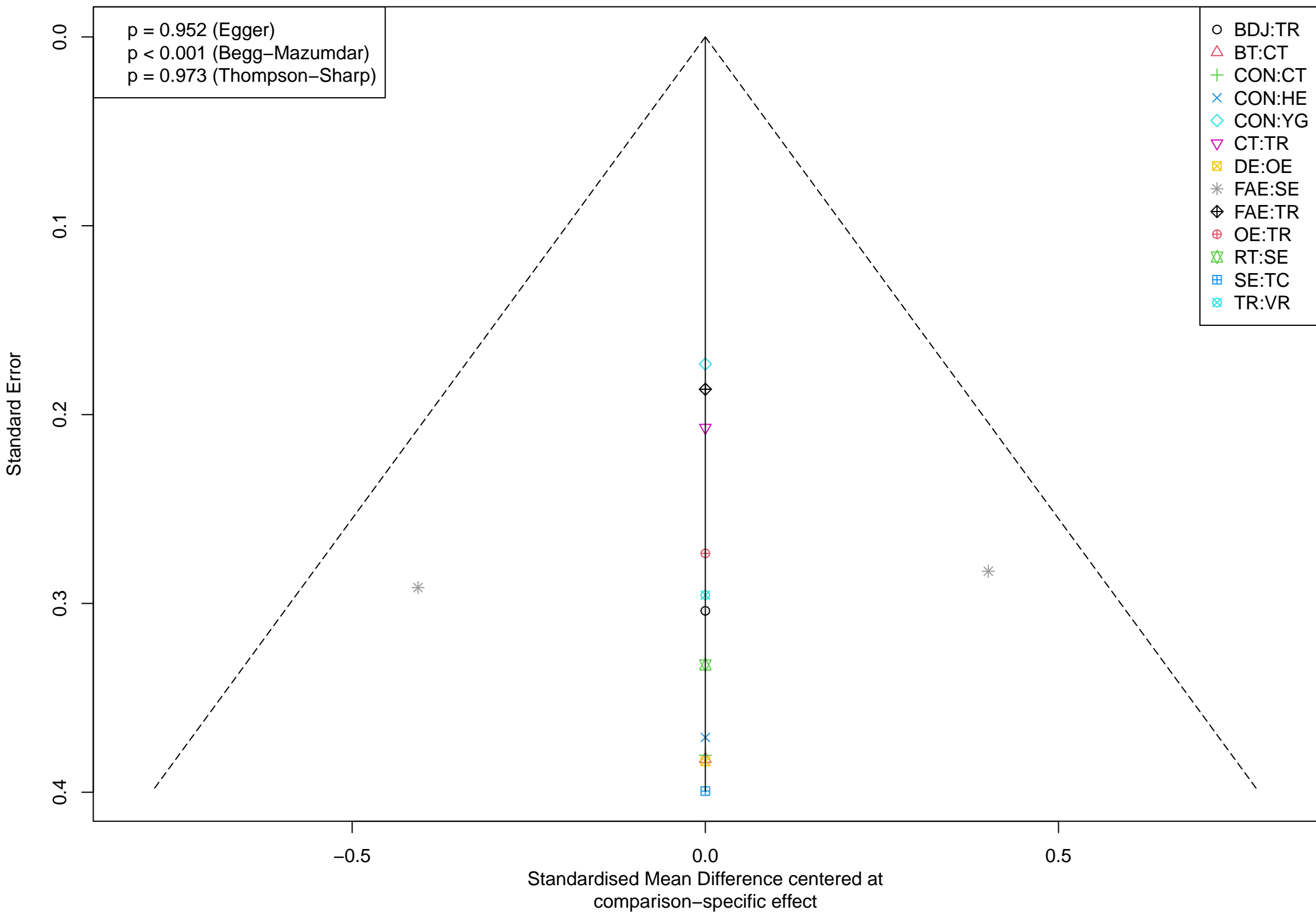

Supplement: Supplementary file 1 [file Data_Sheet_1.zip › Supplementary Material/Appendix 5.2-Funnel polt.pdf]

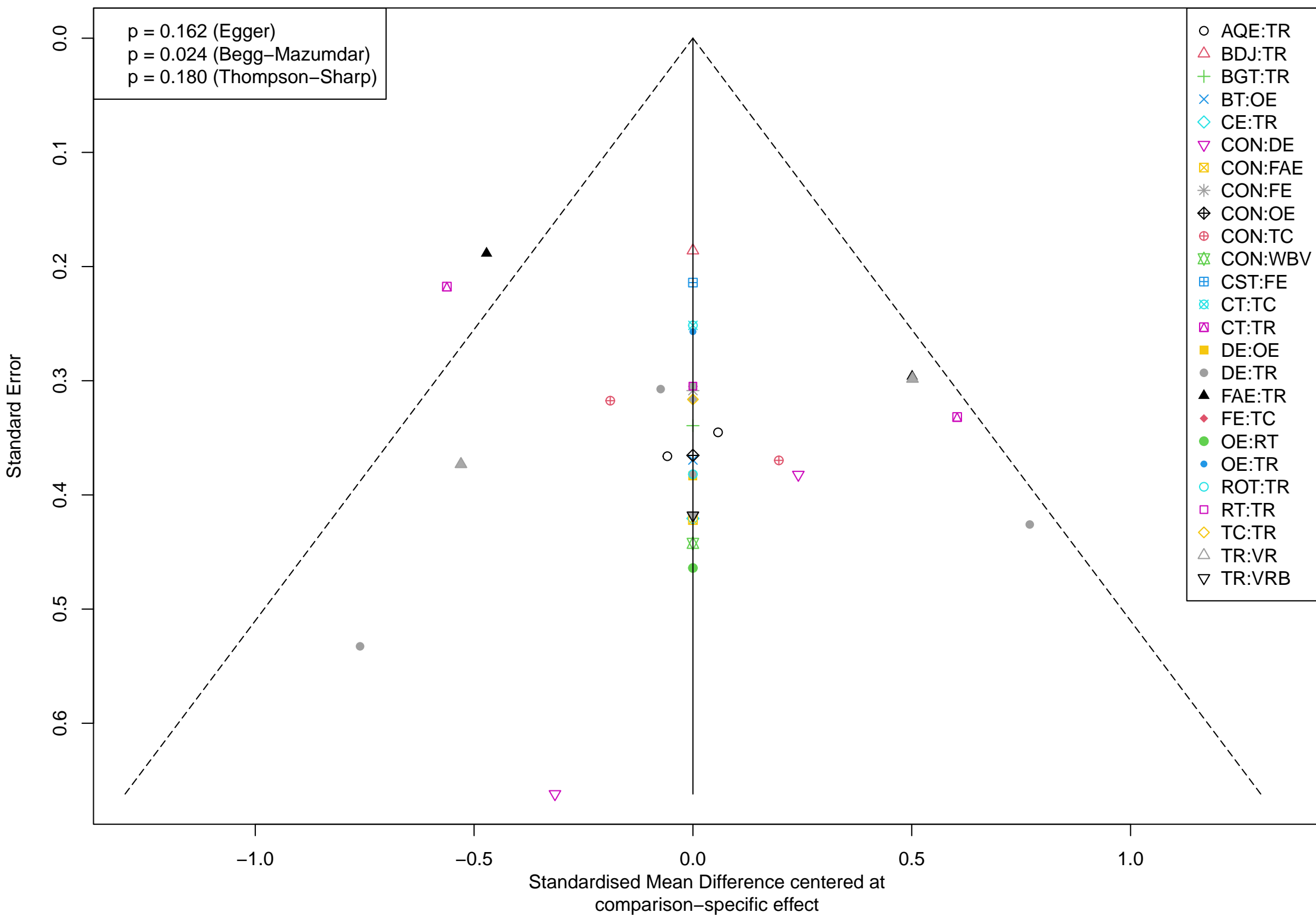

Supplement: Supplementary file 1 [file Data_Sheet_1.zip › Supplementary Material/Appendix 5.3-Funnel polt.pdf]

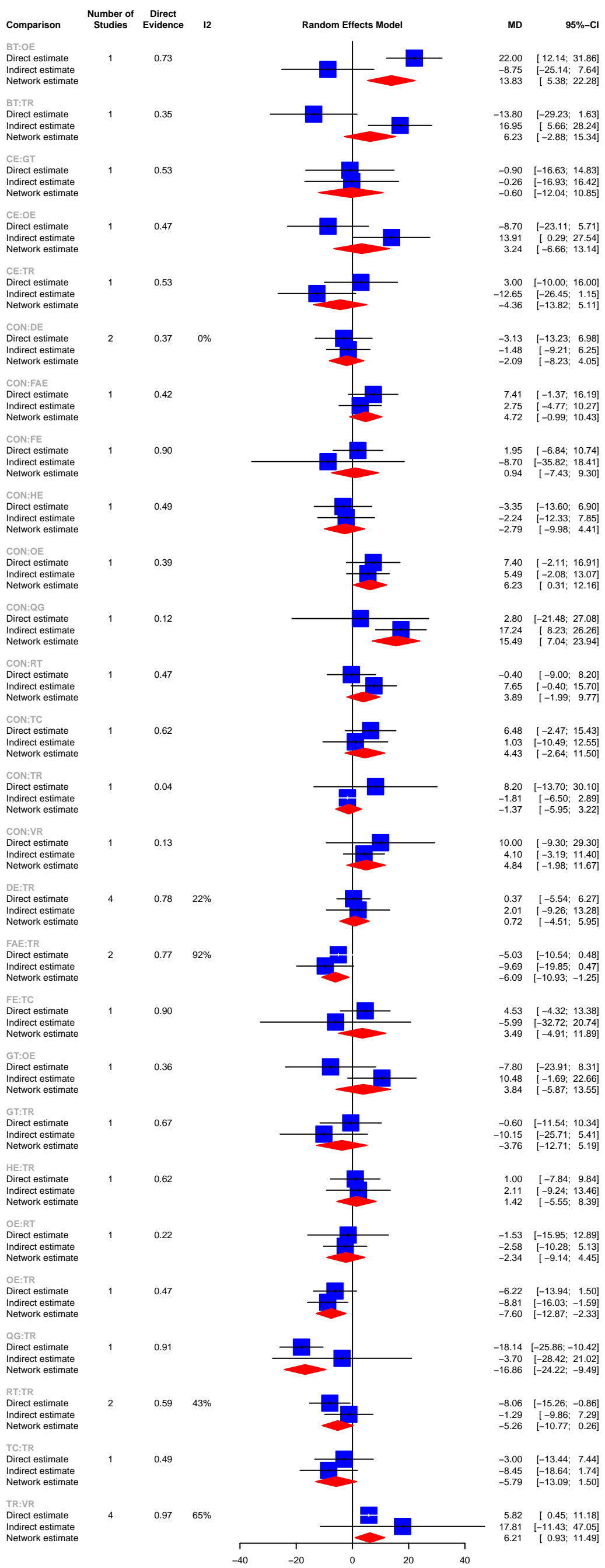

Supplement: Supplementary file 1 [file Data_Sheet_1.zip › Supplementary Material/Appendix 7.1-Node split.pdf]

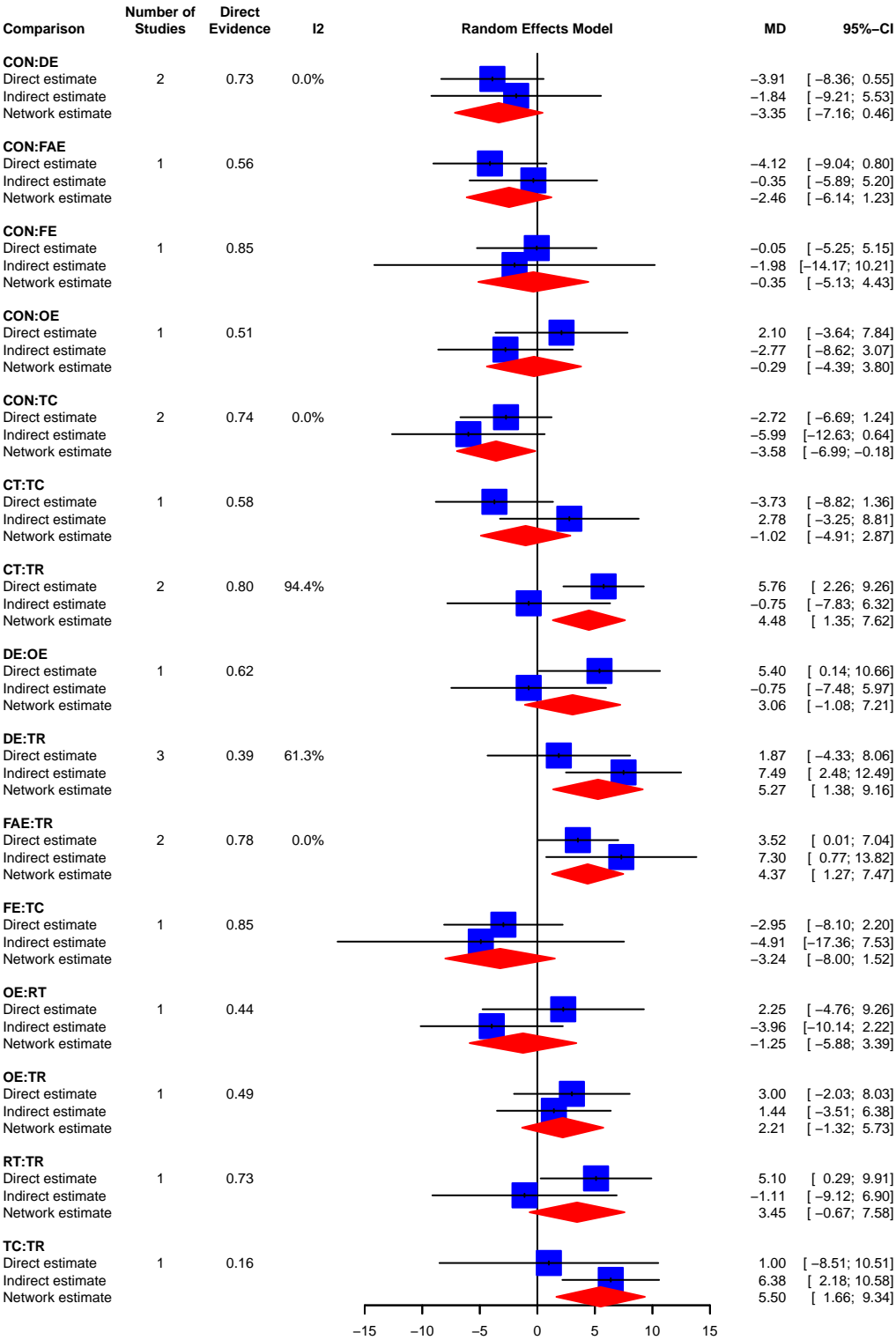

Supplement: Supplementary file 1 [file Data_Sheet_1.zip › Supplementary Material/Appendix 7.2-Node split.pdf]
